# Development of visual predictive checks accounting for multimodal parameter distributions in mixture models

**DOI:** 10.1007/s10928-019-09632-9

**Published:** 2019-04-09

**Authors:** Usman Arshad, Estelle Chasseloup, Rikard Nordgren, Mats O. Karlsson

**Affiliations:** 1 Department of Pharmaceutical Biosciences, Uppsala University, Uppsala, Sweden; 2 Faculty of Medicine and University Hospital Cologne, Center for Pharmacology, Department I of Pharmacology, University of Cologne, Gleueler Str 24, 50931 Cologne, Germany

**Keywords:** Visual predictive checks, Mixture models, Multimodal parameter distributions, Pharmacokinetics, Pharmacodynamics

## Abstract

The assumption of interindividual variability being unimodally distributed in nonlinear mixed effects models does not hold when the population under study displays multimodal parameter distributions. Mixture models allow the identification of parameters characteristic to a subpopulation by describing these multimodalities. Visual predictive check (VPC) is a standard simulation based diagnostic tool, but not yet adapted to account for multimodal parameter distributions. Mixture model analysis provides the probability for an individual to belong to a subpopulation (IP_mix_) and the most likely subpopulation for an individual to belong to (MIXEST). Using simulated data examples, two implementation strategies were followed to split the data into subpopulations for the development of mixture model specific VPCs. The first strategy splits the observed and simulated data according to the MIXEST assignment. A shortcoming of the MIXEST-based allocation strategy was a biased allocation towards the dominating subpopulation. This shortcoming was avoided by splitting observed and simulated data according to the IP_mix_ assignment. For illustration purpose, the approaches were also applied to an irinotecan mixture model demonstrating 36% lower clearance of irinotecan metabolite (SN-38) in individuals with UGT1A1 homo/heterozygote versus wild-type genotype. VPCs with segregated subpopulations were helpful in identifying model misspecifications which were not evident with standard VPCs. The new tool provides an enhanced power of evaluation of mixture models.

**Electronic supplementary material:**

The online version of this article (10.1007/s10928-019-09632-9) contains supplementary material, which is available to authorized users.

## Introduction

Evaluation of the applicability of a model for a specific purpose is a major consideration during pharmacometric analysis. Diagnostic tools have been developed and used extensively for evaluation of pharmacokinetic (PK)/pharmacodynamics (PD) models [1]. The simulation based diagnostic tool known as visual predictive check (VPC) has gathered much focus because of the (i) advantage to retain the original data profile, (ii) ability to describe the central trend and dispersion in the data, and (iii) simplicity for interpretations [2–5]. A VPC is a graphical and statistical comparison of observed and predicted data by deriving the distribution of observations and predictions against the independent variable such as time [3]. Depending on the underlying data, the objective of the study and the intended use of the model, different VPCs such as stratified VPCs (predictive performance across stratification variable such as a covariate), prediction corrected VPCs (to identify random effect misspecification by removing the variability coming from independent variables such as doses) and covariate VPCs (to evaluate the predictive performance of the model across the covariate range) may be used [3, 4].

The nonlinear mixed effect modeling approach quantifies the intrinsic variability associated with pharmacokinetic/pharmacodynamic profiles across the studied population [6]. The underlying assumption of interindividual variability (IIV) being unimodally distributed is not true when the studied population exhibits heterogeneity leading to multimodal parameter distributions [7]. Heterogeneous pharmacological behavior may result in clinically significant differences in drug exposure/toxicity. A classic example involves acetylation polymorphism in case of isoniazid where clearance (CL) was observed to be bimodally distributed and a higher prevalence of peripheral neuropathy and hepatotoxicity was observed in slow metabolizers due to elevated plasma concentrations [8]. Situations may arise where a polymorphism is associated with the exposure/response to a drug, but the covariate capable of describing such behavior is not available. The mixture modeling (also referred as clustering) approach is a useful tool under such circumstances [9]. A number of studies have been reported to utilize mixture modeling. A major proportion of these studies aimed to describe the bimodal distribution of CL as reported in case of serotonin receptor antagonist repinotan, antianginal drug perhexiline and beta-lactam antibiotic ceftizoxime [10–12]. A bivariate absorption describing the subpopulations with and without absorption lag was presented by Piotrovsky et al. [13]. An analysis was performed to segregate the patients with and without adverse effects with the help of adverse event data by Kowalski et al. [14]. Mixture modeling was also applied to model the probability of cure in cancer survival analysis where the proportion of fatal and cured cases was estimated [15–17]. Similarly, a mixture model classifying the mammary tumors in rats as benign or malignant was published by Spilker et al. [18].

Despite the utility of mixture models to describe data arising from a population with underlying heterogeneity, there are limitations in assessing mixture models since the common simulation based assessment tools do not account for the multimodality in parameter distributions. Attempts have been made to develop posterior predictive checks [19] for mixture models [8]. However, VPCs are not yet adapted to mixture models and may fail to adequately evaluate the predictive performance of a mixture model. The aim of the current project was to design VPCs accounting for multimodal parameter distributions and thereby allow (i) the diagnosis of the mixture component aspects of the model, and (ii) more powerful assessment of other model aspects by reducing between-subpopulation variability from the graphs.

## Methods

### Theoretical overview of parameter estimation using mixture models

The underlying assumption behind the mixture modeling approach is to partition the population into subpopulations according to a probability model [8]. With the implementation of mixture models using the $MIXTURE subroutine in NONMEM, pharmacokinetic parameters characteristic to a subpopulation can be obtained [20].

$$CL_{1} = \theta_{1} \times e^{{\eta_{1} }} \quad \ldots clearance\,for\,subpopulation\,1$$

$$CL_{2} = \theta_{2} \times e^{{\eta_{2} }} \quad \ldots clearance\,for\,subpopulation\,2$$

Whereas, the corresponding subpopulation probabilities are estimated as,

$$P_{mix1} = \theta_{3} \quad \ldots probability\,for\, subpopulation\,1$$

$$P_{mix2} = 1 - \theta_{3} \quad \ldots probability\,for\, subpopulation\,2$$

A P_mix1_ estimate of 0.6 corresponds to a 60/40% mixture proportion. The individual likelihood to belong to a subpopulation 1 (IL_mix1_) can be derived from the individual objective function value (IOFV). The individual probability for belonging to a subpopulation (IP_mix_) is then computed from the individual likelihood (IL_mix_) and population probability estimates [7].

$$IL_{mix1} = e^{(IOFV/2)}$$

$$IP_{mix1} = \frac{{IL_{mix1} \times P_{mix1} }}{{IL_{mix1} \times P_{mix1} + IL_{mix2} \times P_{mix2} }}$$

where IL_mix2_ is the corresponding likelihood estimate for the individual to belong to subpopulation 2. The empirical subpopulation assignment that the subject’s data is described by the corresponding submodel is given the name MIXEST within NONMEM.

### Mixture model output

Analysis with mixture models provided two individual-level metrics of subpopulation association (i) the most likely subpopulation for an individual to belong to, and (ii) the probability for an individual to belong to each subpopulation [7]. The former metric (MIXEST) is discrete in nature and can be retrieved from output table files. The latter metric termed IP_mix_ can be retrieved from the *.phm file which is a standard output of models with mixture components. IP_mix_ is considered to be more informative than the MIXEST variable because of its continuous nature.

### Mixture specific VPCs

Two strategies were adapted for allocation of subjects to the subpopulations in order to develop mixture model specific VPCs with separate panels for each allocated subpopulation. The first strategy utilized the MIXEST information to stratify the observed and simulated data. Thus, the original and simulated individuals were separated according to their most likely subpopulation. A tendency for subjects to be allocated to the dominating subpopulation (similar to the shrinkage phenomenon in individual, empirical Bayes, parameter estimation) is expected with the MIXEST-based allocation strategy. This shortcoming was avoided through the second strategy to randomly partition the observed and simulated data according to the IP_mix_ value. Partitioning with the former approach was called MIXEST mixture while the latter was termed randomized mixture. In order to retrieve the IP_mix_ information for the original and simulated data, an evaluation step is required. This was accomplished by directing NONMEM to perform an evaluation step given the final model parameters by setting MAXEVAL = 0 for each simulated data set. Naturally, MIXEST can also be computed from the IP_mix_ value, therefore further processing to derive VPC statistics for graphical display was facilitated by the use of single output file (*.phm). A discrepancy in the individual subpopulation allocation frequency between original and simulated data would be indicative of model misspecification and hence provide an additional evaluation aspect specific for mixture models. Therefore, percentage of individuals in each subpopulation for both the original (ORIGID) and the simulated data (SIMID) and the population estimate for the mixture probability (PMIX) are displayed in the VPC plots.

### Implementation of mixture VPCs

A PsN functionality was developed to direct NONMEM runs and post-processing NONMEM output according to the two strategies (MIXEST and randomized) in order to generate the mixture model VPCs. VPCs were implemented using a ggplot2 based package in R [21–23].

#### Linear PK data

Data was simulated from a one-compartment PK model (ka = 1 h^−1^, CL = 20/80 L/h, V_d_ = 100 L; interindividual variances = 0.09; proportional residual variance = 0.04). A total of 1000 virtual subjects were simulated with 70/30% mixture proportions. Six samples were taken at time points 0.5, 1, 2, 4, 8 and 12 h following a virtual dose of 100 mg. A bivariate covariate resulting in a fourfold difference in CL between subgroups was modeled by the inclusion of a mixture component. In order to compare the mixture model with a model without any mixture component stochastic simulation and estimation (SSE) was performed with PsN version 4.8.0 [24]. The simulated data were analyzed by fitting a covariate-free non-mixture model, a covariate model and a mixture model using NONMEM version 7.4.2 [20]. VPCs were constructed for the mixture model using both the MIXEST and the randomized allocation. Performance of the allocation strategies was evaluated by decreasing the difference in drug CL (20/60 L/h) and increasing size of the dominant subpopulation (85/15% mixture proportions).

#### Parallel linear and nonlinear PK data

Pharmacokinetic data and NONMEM code were extracted from a publically available illustrative PK model example [25]. Thirty-six subjects were part of the analysis with a rich sampling over a period of 672 h (22 observations per individual). Individuals received 4 doses of 50 mg at 0, 168, 336 and 504 h. The pharmacokinetic profile was described by a two-compartment model with two distinct physiological elimination pathways (linear and nonlinear). The pharmacokinetic parameters included V_max_ = 1.2 mg/h, K_m_ = 10 mg/L, CL_linear_ = 0.03/0.12 L/h, V_1_ = 3 L, V_2_ = 2 L and Q = 0.075 L/h. The parameters for drug disposition (CL_linear_, V_max_, V_1_ and V_2_) were scaled with the body weight of each individual. A bivariate covariate describing a fourfolds difference in the linear CL pathway with a 40/60% mixture proportions was introduced before simulation. SSE was performed to simulate the data given the model parameters followed by estimation with a mixture model. Mixture specific VPCs were developed to assess the predictive performance of the model.

#### Irinotecan PK data

Irinotecan PK profile was described by a combined model from previously published studies [26, 27]. Data comprised of 109 patients with various malignant solid tumors who received an intravenous infusion of 100–350 mg/m^2^ for a period of 0.75–2.25 h. A total number of 1930 plasma concentration measurements of active metabolite SN-38 were available for the analysis. The model (Fig. 5) comprised of a three-compartment model for the parent drug, a two-compartment model for the active metabolite (SN-38) and a two-compartment model for the inactive glucuronide conjugate of SN-38 (SN-38G). The drug was characterized by linear PK properties and the disposition parameters were scaled with body surface area. IIV was associated with all the parameters and the residual unexplained variability was modeled by an additive model. Based on the established influence of genetic polymorphism upon SN-38 CL, a mixture model was developed as the patient genotype information was unavailable. Traditional and mixture specific VPCs were developed for the irinotecan mixture model for comparative evaluation of the recently developed methodology.

## Results

**Fig. 1:**
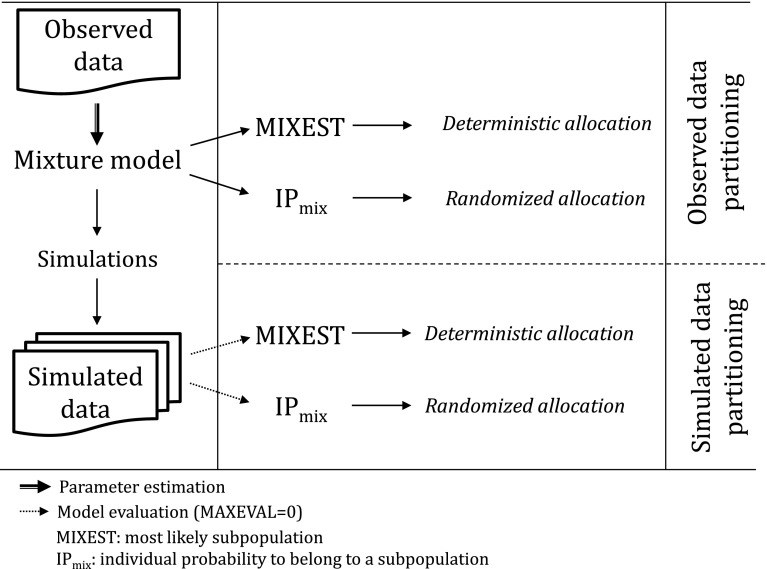
Illustration of proposed methodology

Allocation of individuals to subpopulations according to MIXEST and IP_mix_ information is elaborated in Fig. 1.

### VPCs for linear PK data

**Fig. 2:**
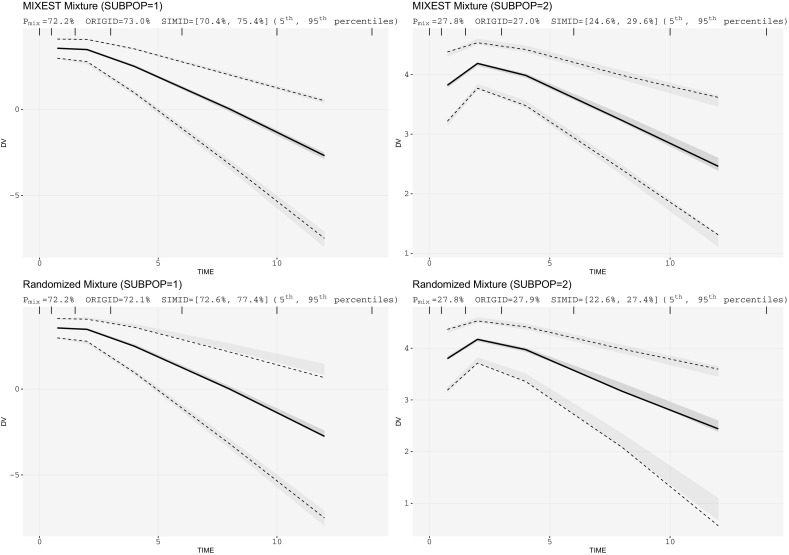
Mixture VPCs for linear PK data: upper panel displays MIXEST based VPCs while lower panel displays IP_mix_ based VPCs. One-compartment mixture model with 70/30% mixture proportions having fourfolds CL difference. (*SUBPOP* subpopulation number, *P*_*mix*_ estimated population proportion, *ORIGID, SIMID* individuals (%) allocated to respective subpopulations in original and simulated data respectively)

**Fig. 3:**
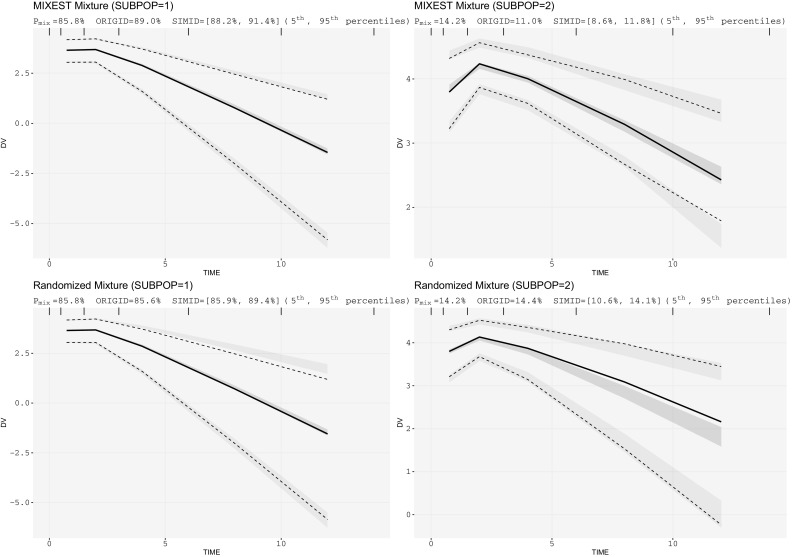
Mixture VPCs for parallel linear and nonlinear PK data: one-compartment mixture model with 85/15% mixture proportions having threefolds CL difference

SSE results showed that the mixture model with a P_mix_ estimate of 72.2% provided an improved goodness-of-fit (OFV = − 664) over the covariate free, non-mixture model (OFV = − 642). The inclusion of covariate information provided the best fit (OFV = − 774), as expected. Figure 2 presents the mixture specific VPCs for the simulated PK data with linear kinetics. Both the MIXEST and the randomized mixtures were adequate to evaluate the predictive performance of the model. However, for a population with a comparatively lower difference in drug CL (20/60 L/h) and a greater proportion of dominant subpopulation (P_mix_ estimate of 85.8%) an allocation bias towards the dominant subpopulation was observed with the MIXEST based method (Fig. 3).

### VPCs for parallel linear and nonlinear PK data

**Fig. 4:**
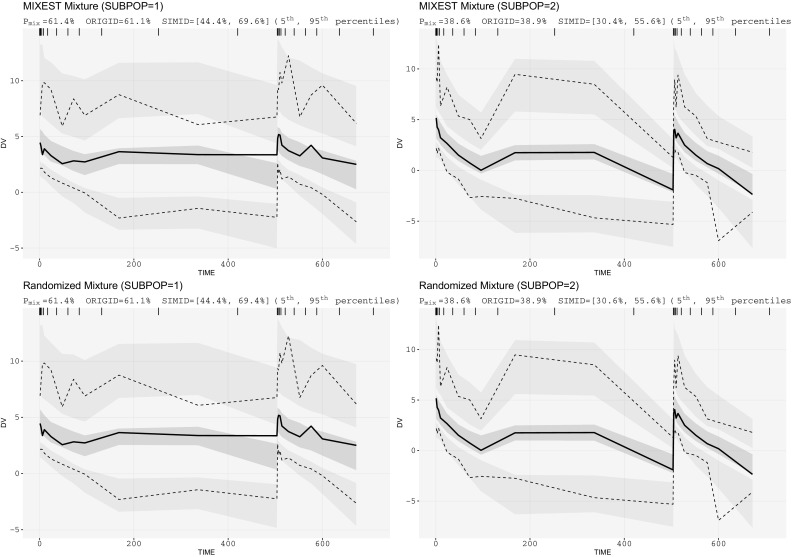
Mixture VPCs for irinotecan PK data: two-compartment model with mixed elimination kinetics having a mixture proportion of 60/40% with fourfolds CL difference (mixture component on linear CL model)

Mixture VPCs for a mixture model describing parallel linear and nonlinear CL pathways are presented in Fig. 4. P_mix_ was estimated to 61.4%. No allocation bias was observed in this case as the fourfolds difference in CL for the linear pathway was sufficient to separate the subpopulations with 40/60% proportions.

### VPCs for irinotecan PK data

**Fig. 5:**
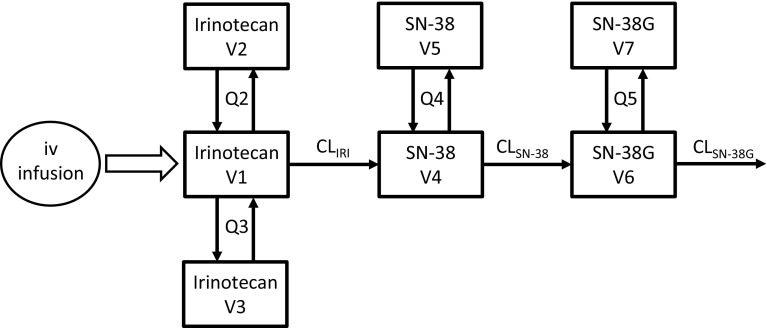
Schematic representation of the irinotecan mixture model having 36% lower CL of SN-38 in patients with UGT1A1 hetero/homozygote versus wild-type genotype

**Fig. 6:**
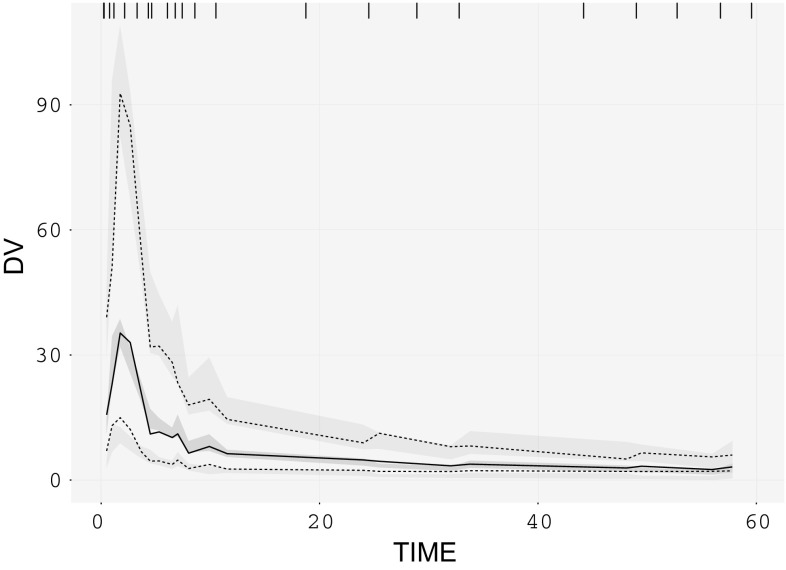
Traditional VPC for irinotecan mixture model

**Fig. 7:**
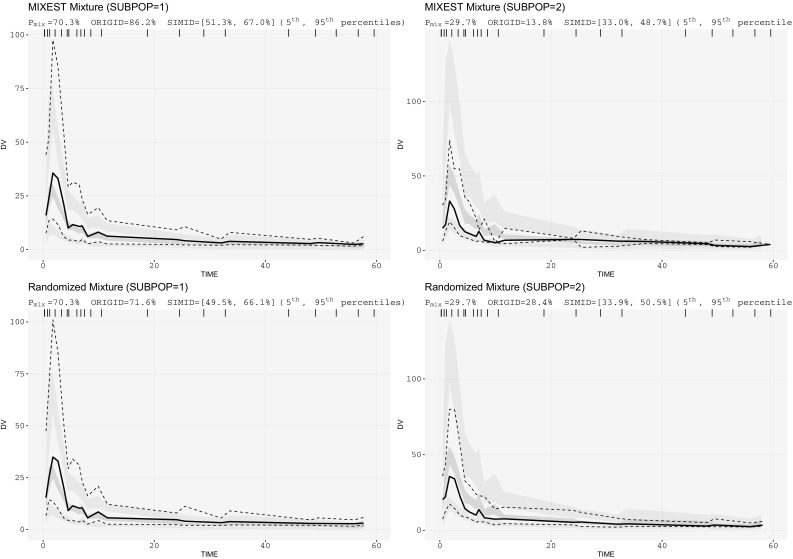
Mixture VPCs for irinotecan mixture model; left panel: VPCs for slow metabolizers; right panel: VPCs for fast metabolizers

The irinotecan mixture model (Fig. 5) provided a P_mix_ estimate of 70.3% and an approximately 36% lower CL of SN-38 in patients with UGT1A1 hetero/homozygote (*1/*28, *28/*28) versus wild-type (*1/*1) genotype. The traditional VPC (Fig. 6) did not show any model misspecification implying that the model was adequate to describe the pharmacokinetics of the population under study. However, a model misspecification was captured with the implementation of recent approaches. It was evident from mixture VPCs (Fig. 7) that the mixture model was under-predictive for slow metabolizers while over-predictive for fast metabolizers.

## Discussion

A major objective during population analysis is to identify, or otherwise manage, the sources of variability in order to assist decision making. Sources for variability characterized in PK/PD models result in predictable differences in exposure/responses between patient groups and provide a tool to tailor the treatment individually. Identifying not only the magnitude, but also the shape of the unexplained variability can be important. Mixture models are suitable for appropriately characterizing multimodality associated with parameter distributions. VPC is considered to be one of the most informative tools, able to simultaneously diagnose the fixed and random effects [3, 4]. Therefore, mixture VPCs were designed to overcome the limitations of the classical VPCs for the evaluation of mixture models.

Evaluation with the two VPC implementation strategies for simulated data (Fig. 2) illustrates how mixture VPCs can be useful to split the data into subpopulations thereby enhancing the power of evaluation by decreasing the remaining variability within a subpopulation. Both the MIXEST and the IP_mix_ based allocation strategies were adequate to cluster the simulated data for a drug exhibiting linear PK with sufficiently differentiable CL (20/80 L/h). Apart from the visual evaluation, information provided in the display is of significant importance. The population probability estimate (P_mix_) is representative of the agreement of the model with prevalence of subpopulations in existing literature. Uncertainty or bias associated with P_mix_ can be reflective of model misspecification or insufficient information available in the data. The number of individuals allocated to the respective subgroups should be in accordance with the P_mix_ estimate. Allocation bias in the original and the simulated data can be evaluated from the values assigned to ORIGID and SIMID. No discrepancy between MIXEST and IP_mix_ based allocation of individuals in this illustrative example implies that the data was informative enough to separate the individuals according to their likelihood/probability estimates.

As multimodal parameter distributions stem from a failure to incorporate a multimodally distributed covariate in the model, it is good practice to consider existing covariate data before the decision to proceed with mixture models. Model comparison using SSE results confirm that the covariate model provides a preference over the mixture model, while a mixture model in turn is a better characterization of the data compared to the standard, unimodal distribution.

**Fig. 8:**
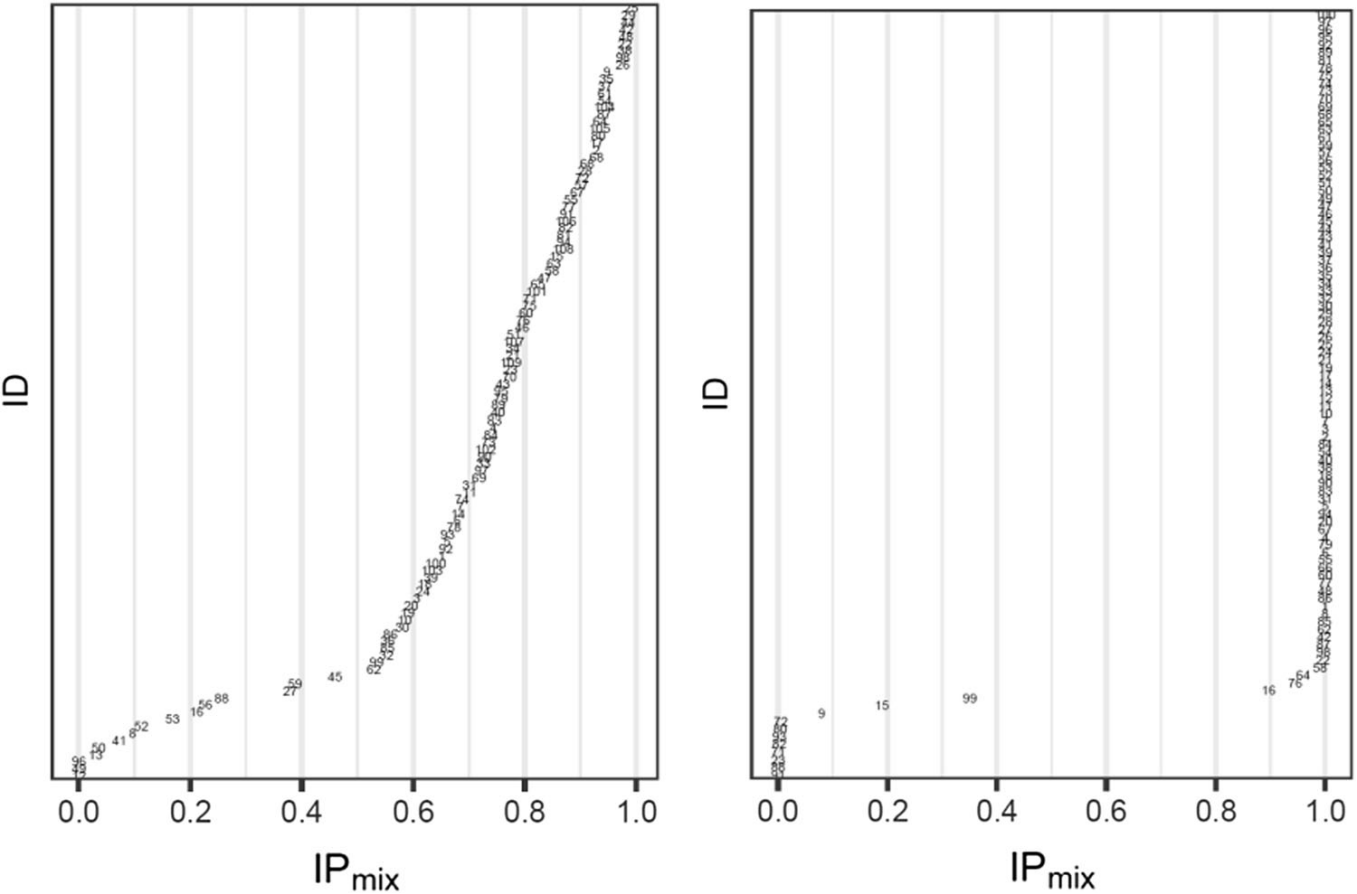
Distribution of individuals in a population; left panel: a less separated mixture; right panel: a clearly separated mixture

Under circumstances where the individual data is less informative, the MIXEST estimate may exhibit shrinkage towards the dominant subpopulation in contrast to IP_mix_. Kaila et al. [28] used Monte Carlo simulations to examine factors that might impact the ability to correctly classify a subject in a bimodal group. Using a one-compartment model with subjects assigned to one of two CL groups, the authors found that misclassification of individuals was dependent on (i) the magnitude of the difference between the mean CL estimates for the subgroups, (ii) IIV in CL, (iii) proportion of subjects in each subpopulation and (iv) sample size. One should be careful to inspect multimodalities in all the parameter estimates and not only the parameter of physiological interest. A probability partitioning may exist across more than one parameter. There may be a 30/70% partitioning for CL, but a 10/90% partitioning for the volume of distribution. Analysis of such data with a model containing a single mixture component may also lead to uncertainty in probability estimates leading to misclassification or biased allocation. Figure 3 demonstrates a biased allocation where the fraction of the dominant subpopulation was larger (85/15%) and the difference in CL was comparatively lower (20/60 L/h). An allocation bias of 3.2% towards the larger subpopulation can be observed with the MIXEST mixture. The less informative individuals with IP_mix_ estimate around 0.5 can be identified with the help of a diagnostic plot displaying the distribution of individuals in a mixture (Fig. 8). The plot presents a less separated (left) and a clearly separated (right) mixture population. We hereby demonstrate that a randomized allocation based upon IP_mix_ information takes into account the uncertainty for an individual to belong to a subpopulation where the data from an individual is less informative.

Figure 4 displays VPCs for a population with mixed elimination kinetics. The phenomenon is often observed for therapeutic monoclonal antibodies. The linear CL pathway is possibly mediated by antibody Fc-receptors interaction, while the nonlinear CL pathway reflects binding to its pharmacologic target. A higher allocation bias (16%) using MIXEST method was observed with the evaluation of irinotecan mixture model. Moreover, a clear model misspecification was observable from mixture VPCs (Fig. 7) which was otherwise not evident from the classical VPC (Fig. 6). Irinotecan mixture VPCs were supportive of the argument that by reducing the between subpopulation variability in the VPC an enhanced power of evaluation can be achieved. Mixture VPCs were suggestive of further structural model modifications to adequately describe the subpopulation profiles but the respective analysis was beyond the scope of current project.

VPCs like other simulation-based diagnostics test a model’s ability to generate data that mimics the observed data. Systematic differences between simulated and real data indicate the deficiency of the model to predict the observed data. An important aspect regarding such procedures is that post-processing of both the observed and simulated data is done in similar way, regardless of whether the post-processing occurs through model-based or model-independent methods. Indeed, model-based post-processing can be advantageous to learn about the model misspecifications [29, 30]. Capturing misspecification in a feature of the model does not necessarily mean that the model is inadequate for its purpose. Such decisions are contextual in nature. Although, a considerable number of cases can be seen where mixture modeling approach was used to report results [31–40], but the class of mixture models did not gather much attention to develop diagnostics. Recommended diagnostics for the assessment of non-linear mixed effects models such as VPC, conditional weighted residuals (CWRES), normalized prediction distribution errors (NPDE) are relatively new [1] and less applicable to mixture models. A recent procedure was presented by Lavielle et al. [41] but does not address mixture models either. Implementation of recent methodology would assist both model developers and users to better assess the mixture aspects than what is being practiced currently.

The proposed methodologies are implemented in PsN and VPCs can be generated with the addition of the option −mix to the vpc command. For comparative evaluation purpose, a traditional VPC plot was also included in the PsN output.

## Conclusions

A graphical and statistical comparison of observations and predictions derived from the multimodal distributions in mixture models is presented. Partitioning of observed and predicted data between subpopulations can be done in two ways depending on the underlying information (MIXEST or IP_mix_). Randomized allocation based upon individual IP_mix_ information provides a preference over MIXEST based discrete allocation as a lower allocation bias is associated with the former case. Mixture VPCs can be a useful diagnostic tool for the development and evaluation of mixture models in the future.

## Electronic supplementary material

Below is the link to the electronic supplementary material.

Supplementary material 1 (RMD 2 kb)

Supplementary material 2 (R 3 kb)
